# Cell-free microRNA-1246 in different body fluids as a diagnostic biomarker for esophageal squamous cell carcinoma

**DOI:** 10.1371/journal.pone.0248016

**Published:** 2021-03-10

**Authors:** Isamu Hoshino, Fumitaka Ishige, Yosuke Iwatate, Hisashi Gunji, Naoki Kuwayama, Yoshihiro Nabeya, Hajime Yokota, Nobuyoshi Takeshita, Keiko Iida, Hiroki Nagase, Hisahiro Matsubara

**Affiliations:** 1 Division of Gastroenterological Surgery, Chiba Cancer Center, Chiba, Japan; 2 Department of Hepatobiliary and Pancreatic Surgery, Chiba Cancer Center, Chiba, Japan; 3 Department of Diagnostic Radiology and Radiation Oncology, Graduate School of Medicine, Chiba University, Chiba, Japan; 4 Division of Surgical Technology, National Cancer Center Hospital East, Chiba, Japan; 5 Department of Frontier Surgery, Graduate School of Medicine, Chiba University, Chiba, Japan; 6 Laboratory of Cancer Genetics, Chiba Cancer Center Research Institute, Chiba, Japan; Taipei Medical University College of Medicine, TAIWAN

## Abstract

Esophageal squamous cell carcinoma is a malignant tumor with unfavorable prognosis. In this study, we investigated the usefulness of microRNA (miR)-1246 detection in various body fluids as a biomarker for this disease. A total of 72 patients with esophageal squamous cell carcinoma were enrolled, and their blood, urine, and saliva samples were collected prior to treatment. Reverse transcription–polymerase chain reaction of miR-1246 was performed, and pre- and postoperative and intraday fluctuations in its expression were examined. The expression of miR-1246 in the blood and urine was significantly higher in the patients with esophageal squamous cell carcinoma than in 50 healthy control subjects. Receiver operating characteristic curves showed that the area under the curve values were 0.91 (sensitivity 91.7%, specificity 76.0%), 0.82 (sensitivity 90.3%, specificity 62.0%), and 0.80 (sensitivity 83.3%, specificity 66.0%) in the serum, urine, and saliva, respectively. A relatively high diagnostic performance of miR-1246 was observed in all samples, which was better than that of the existing biomarkers squamous cell carcinoma antigen, carcinoembryonic antigen, and cytokeratin 19 fragment. No clear correlation was observed in the levels of miR-1246 expression among the three body fluids. Postoperatively, serum samples displayed significantly decreased miR-1246 levels. Although not significant, changes in the miR-1246 levels were observed at all collection times, with large fluctuations in the saliva. Meanwhile, serum miR-1246 expression was found to be associated with the disease prognosis. The results indicate that the levels of miR-1246 in the urine, saliva, and serum are a useful biomarker for esophageal squamous cell carcinoma and support the use of urine samples instead of blood samples for noninvasive diagnosis.

## Introduction

Esophageal cancer is a common gastrointestinal malignancy, with 572,000 cases and 509,000 deaths reported globally in 2018 [1]. There are two major pathological categories of esophageal cancer, namely, esophageal squamous cell carcinoma (ESCC) and adenocarcinoma, and the former is the major histological type throughout Asia [2]. Smoking and alcohol intake are causative factors for ESCC. At the molecular level, recent studies have revealed that approximately 59–93% of patients with ESCC harbor mutations in tumor protein p53 [3]. With current clinical resources, the ESCC prognosis is poor, with an overall 5-year survival rate of 20–30% [4]. When disease is detected early, the 5-year survival rate for patients with ESCC is 80–90% [5,6]; however, early-stage esophageal cancer is less likely to show clinical symptoms, and a lack of reliable noninvasive screening methods hinders its detection. Therefore, the establishment of diagnostic markers for ESCC is crucial to improve patient survival [7,8].

MicroRNAs (miRs) are very short (19–22 bases) non-coding RNAs. In 2005, a study reported that miRs could classify tumors more accurately than mRNA expression profiles could [9]. Since then, researchers have mainly focused on the potential use of miRs as blood-based diagnostic biomarkers for cancer [10–12]. However, attempts have recently been made to detect miRs in other body fluids, such as urine and saliva, as simpler, noninvasive options [13–18].

Previously, we have focused on the expression of miRs in the serum of patients with esophageal cancer, and a comprehensive analysis indicated that miR-1246 was the most highly expressed miR [19]. The results of PCR using other samples also showed that miR-1246 was significantly upregulated in patients with ESCC compared with that in healthy subjects and was reported to be useful as a novel biomarker for esophageal cancer. It was also found that patients with high levels of miR-1246 expression in the serum had a poorer prognosis than that of patients with low miR-1246 expression. miR-1246 functions as part of the p53-related intercellular network and has been reported to be associated with the resistance to cancer chemotherapy and with cancer stem cell-like properties [20,21]. Furthermore, in our previous report, miR-1246 expression levels were elevated not only in the serum of patients with pancreatic cancer but also in the urine and saliva [22].

In this study, we quantitatively and simultaneously measured miR-1246 expression in the serum, urine, and saliva of patients with ESCC and healthy controls and examined the clinical significance of the findings.

## Materials and methods

### Ethical approval

Written informed consent was obtained from all patients, and the study was approved by the Ethics Committee of the Chiba Cancer Center (No. H29-0005) and performed in compliance with the principles of the Declaration of Helsinki.

### Samples

Between April 2017 and October 2020, venous blood, urine, and saliva samples were collected from 72 patients with ESCC and 50 healthy controls at the Chiba Cancer Center in Chiba, Japan. Samples were collected before any treatment, including endoscopic resection, surgery, chemotherapy, and radiotherapy. Postoperative samples were obtained from 10 patients 3 weeks after surgery. Venous blood samples were centrifuged at 1,500 × *g* for 5 min at 4°C to obtain serum. Urine collection was performed when it was convenient for the patient, and saliva collection was performed, after an oral rinse, at any time other than immediately after a meal. Urine and saliva samples were centrifuged at 1,500 × *g* for 5 min at 4°C to obtain supernatants. If saliva separation was incomplete, it was centrifuged for an additional 5 min. The samples were then stored at −80°C until further processing.

### RNA extraction

Total RNA was extracted from 200 μL of serum, urine, and saliva using the miRNeasy serum/plasma kit (Qiagen, Hilden, Germany) according to the manufacturer’s instructions. This kit contains *Caenorhabditis elegans* cel-miR-39, which was used as a spike-in control.

### Reverse transcription

Total RNA was reverse transcribed to cDNA using the miScript II RT kit (Qiagen). In each reaction, 50 ng (12 μL) of template RNA was combined with a master mix containing 4 μL of 5× miScript HiSpec buffer, 2 μL of 10× miScript Nucleics mix, and 2 μL of miScript reverse transcriptase mix. The reactions were incubated for 60 min at 37°C, followed by incubation for 5 min at 95°C to inactivate reverse transcriptase, and then placed on ice.

### Quantitative RT-PCR

Quantitative RT-PCR was performed using the miScript SYBR® Green PCR kit (Qiagen) in a 7300 real-time PCR system (Applied Biosystems, Foster City, CA, USA). The sequences of the forward primers used for miR-1246 and cel-miR-39 were 5′-AAUGGAUUUUUGGAGCAGG-3′ and 5′-UCACCGGGUGUAAAUCAGCUUG-3′, respectively. The parameters of RT-PCR were as follows: 95°C for 15 min, followed by 40 cycles of 94°C for 15 s, 55°C for 30 s, and 70°C for 34 s. All reactions were performed in duplicate. Relative expression was calculated using comparative cycle threshold (Ct) values. Relative miR-1246 expression was calculated using the 2^−ΔCt^ method, where ΔCt = Ct (miR-1246) − Ct (cel-miR-39).

### Statistical analysis

Normal distribution of data was confirmed using the Shapiro–Wilk test. An unpaired Student’s *t*-test was performed to compare differences in age. Wilcoxon’s signed-rank test was performed to compare differences in miR-1246 expression levels between patients with cancer and healthy controls. Spearman’s rank correlation coefficient was used to assess correlations among miR-1246 expression levels in the three body fluids. The χ^2^ test or Fisher’s exact probability test was used to evaluate correlations between serum and urine miR-1246 expression levels and clinicopathological tumor factors. Receiver operating characteristic (ROC) curves and areas under the curves (AUCs) were used to assess the sensitivity and specificity of serum, urine, and saliva miR-1246 expression levels in detecting ESCC. All tests were two-sided, and the significance level was set at a p-value < 0.05. The survival period of the patients was defined as the duration from the time of surgery to either death or the last follow-up, and the survival rate was calculated using the Kaplan–Meier method. Comparisons of two groups in univariate analyses were performed using the log-rank test. To evaluate the diurnal variation of miR-1246 in each body fluid, the intraclass correlation coefficient (ICC) was calculated using a two-way random model. The ICC values were interpreted as follows: ≤ 0.20, a slight agreement; 0.21–0.40, a fair agreement; 0.41–0.60, a moderate agreement; 0.61–0.80, a substantial agreement; and 0.81–1.00, a nearly complete agreement [23]. The JMP 14 software (SAS Institute, Inc., Cary, NC, USA) was used for all analyses.

## Results

### Patient details and miR-1246 expression levels in each body fluid

**Fig 1:**
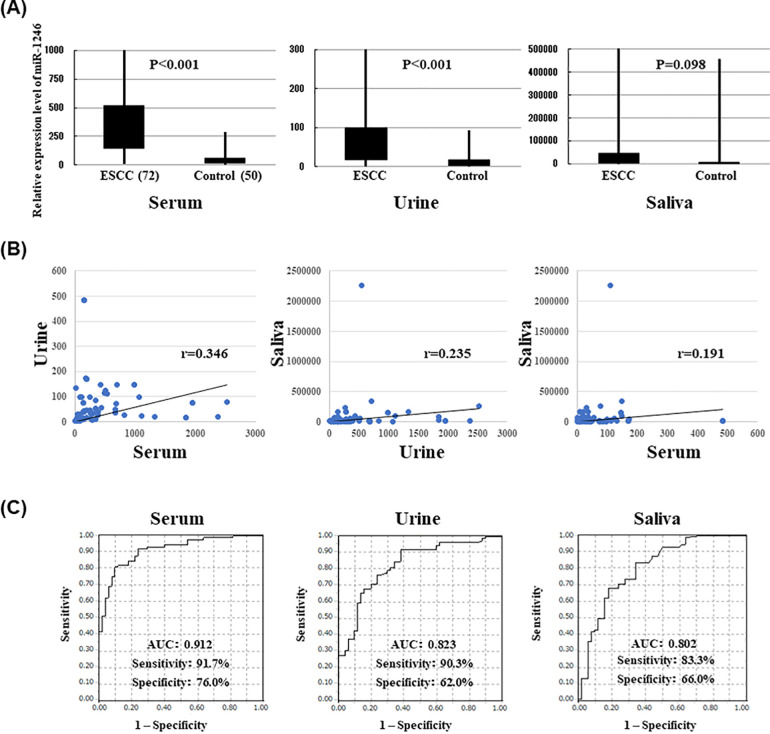
A. Serum, urine, and saliva samples were collected from 72 patients with ESCC and 38 healthy controls. The expression level of miR-1246 was evaluated using RT-PCR. It was confirmed that the expression level of miR-1246 was significantly higher in serum and urine in patients with ESCC than in controls. On the other hand, the expression level of miR-1246 in saliva tended to be higher in the patient group, but it was not significant. B. The correlation of the expression level of miR-1246 in each body fluid was confirmed. No clear correlation was observed between the levels of miR-1246 expression in the three body fluids. C. The results of the ROC curve analysis of the miR-1246 expression levels in each body fluid. The AUC was 0.912 (sensitivity 91.7%, specificity 76.0%) for serum miR-1246, 0.823 (sensitivity 90.3%, specificity 62.0%) for urine miR-1246, and 0.802 (sensitivity 83.3%, specificity 66.0%) for saliva miR-1246.

The patient details are shown in Table 1. The expression levels of miR-1246 in the serum, urine, and saliva of the patients with ESCC (n = 72) were compared with those of the healthy controls (n = 50). The serum and urine miR-1246 expression levels were significantly higher in the patients with ESCC than in the healthy controls (p < 0.001). The expression of miR-1246 also tended to be higher in the saliva of the patients with ESCC, but the difference was not significant (p = 0.098; Fig 1A). No clear correlation was observed among the levels of miR-1246 expression in the three body fluids (r < 0.50, p < 0.001; Fig 1B).

**Table 1:** Patient details and clinicopathological features.

	Esophageal cancer	Healthy control	p value
**Number**	72	50	
**Gender**			
Male	65 (90.3)	42 (84.0)	0.448
Female	7 (9.7)	8 (16.0)	
**Mean age ± s.d. (years)**	70.4 ± 8.8	64.5 ± 9.9	0.119
**Age range (years)**	47–88	41–81	
**Smoking**			
Yes	58 (80.6)	33 (66.0)	0.109
No	14 (19.4)	17 (34.0)	
**Drinking**			
Yes	63 (80.6)	41 (66.0)	0.560
No	9 (19.4)	9 (34.0)	
**Depth of tumor invasion**			
T1	29 (40.3)		
T2	10 (13.9)		
T3	26 (36.1)		
T4	7 (9.7)		
**Lymph node metastasis**			
Positive	30 (41.7)		
Negative	42 (58.3)		
**Distant metastasis**			
Positive	3 (4.2)		
Negative	69 (95.8)		
**TNM stage**			
I	25 (34.7)		
II	20 (27.8)		
III	22 (30.6)		
IV	5 (6.9)		
**Mean miR-1246 expression ± s.d.**			
Serum	474.2 ± 569.6	54.6 ± 63.0	<0.001
Urine	74.4 ± 99.0	16.1 ± 23.4	<0.001
Saliva	73220.8 ± 269903.9	17611.2 ± 68672.6	0.098

### Diagnostic capacity of miR-1246 in each body fluid

ROC curve analysis revealed the sensitivity of miR-1246 levels as a diagnostic indicator of ESCC in each body fluid (Fig 1C). The AUC was 0.912 (sensitivity 91.7%, specificity 76.0%) for serum miR-1246, 0.823 (sensitivity 90.3%, specificity 62.0%) for urine miR-1246, and 0.802 (sensitivity 83.3%, specificity 66.0%) for saliva miR-1246.

### Positive detection rates using miR-1246 levels in various body fluids and those of conventional tumor markers

**Fig 2:**
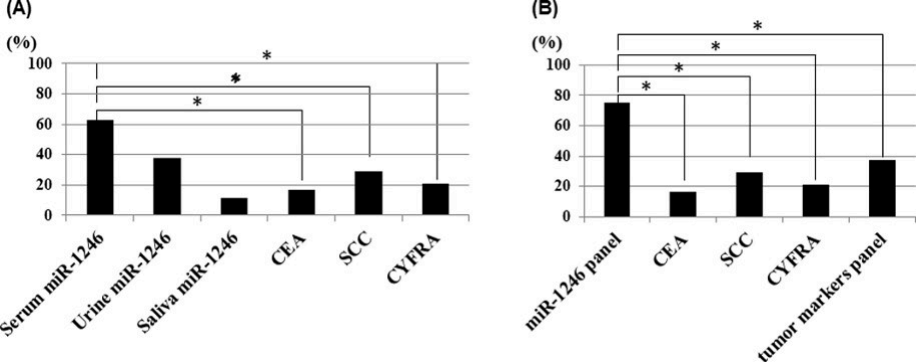
Sensitivity of miR-1246 in each body fluid and conventional tumor markers carcinoembryonic antigen (CEA), squamous cell carcinoma antigen (scc), and CYFRA. A, The sensitivities of the serum, urine, and saliva miR-1246 levels to detect ESCC were 62.5%, 37.5%, and 11.1%, respectively. The sensitivities of serum carcinoembryonic antigen, squamous cell carcinoma antigen, and CYFRA were 16.7%, 29.2%, and 20.8%, respectively. B, The sensitivity of all miR-1246 fluid levels combined was 75.0%, while the combined sensitivity of the conventional tumor markers was 37.5%. Panels combining miR-1246 levels in each body fluid had significantly higher positive rates than individual existing tumor markers or panels combining them. (*:p<0.001).

Using the mean miR-1246 expression level plus two standard deviations in the control group as a threshold, the sensitivities of the serum, urine, and saliva miR-1246 levels to detect ESCC were 62.5%, 37.5%, and 11.1%, respectively. The corresponding sensitivities of serum carcinoembryonic antigen, squamous cell carcinoma antigen, and cytokeratin 19 fragment (CYFRA) were 16.7%, 29.2%, and 20.8%, respectively (Fig 2A). The sensitivity of all miR-1246 fluid levels combined was 75.0%, while the combined sensitivity of the conventional tumor markers was 37.5% (Fig 2B). Panels combining miR-1246 levels in each body fluid had significantly higher positive rates than individual existing tumor markers or panels combining them.

### Relationships between miR-1246 levels in each body fluid and clinicopathological factors of ESCC

Statistical analysis was performed to determine the existence of relationships between serum, urine, and saliva miR-1246 levels and clinicopathological factors of ESCC (Table 2). Patient samples were divided at their median miR-1246 expression levels to obtain high- and low-expression groups. Consistent with our previous report [19] high serum miR-1246 expression showed a tendency to correlate with tumor invasion and positive lymph node metastasis, albeit insignificantly. The expression levels of miR-1246 in the urine and saliva were not related to any clinicopathological factor.

**Table 2:** The correlation between the 1246 and clinicopathological features of ESCC.

characteristics	n	High miR-1246 in serum	Low miR-1246 in serum	P-value	High miR-1246 in urine	Low miR-1246 in urine	P-value	High miR-1246 in saliva	Low miR-1246 in saliva	P-value
total (%)	72	36 (50.0)	36 (50.0)		36 (50.0)	36 (50.0)		36 (50.0)	36 (50.0)	
**Sex**										
Male (%)	65	32 (44.4)	33 (45.8)	1	33 (45.8)	32 (44.4)	1	34 (48.2)	31 (43.1)	0.426
Female (%)	7	4 (5.6)	3 (4.2)		3 (4.2)	4 (5.6)		2 (1.8)	5 (6.9)	
**Age**										
<65 (%)	14	6 (8.3)	8 (11.1)	0.766	9 (12.5)	5 (6.9)	0.372	6 (8.3)	8 (11.1)	0.766
65≦ (%)	58	30 (41.7)	28 (38.9)		27 (37.5)	31 (43.1)		30 (41.7)	28 (38.9)	
**Tumor depth**										
T1-2 (%)	39	15 (20.8)	24 (33.3)	0.058	20 (27.8)	19 (26.4)	1	19 (26.4)	20 (27.8)	1
T3-4 (%)	33	21 (29.2)	12 (16.7)		16 (22.2)	17 (23.6)		17 (23.6)	16 (22.2)	
**Lymph node metastasis**										
Negative (%)	42	17 (23.6)	25 (35.7)	0.094	21 (29.2)	21 (29.2)	1	22 (30.6)	20 (27.8)	0.633
Positive (%)	30	19 (26.4)	11 (35.7)		15 (20.8)	15 (20.8)		14 (19.4)	16 (22.2)	
**Metastasis**										
Negative (%)	69	33 (45.8)	36 (50.0)	0.238	34 (47.2)	35 (48.6)	1	34 (47.2)	35 (48.6)	1
Positive (%)	3	3 (4.2)	0 (0.0)		2 (2.8)	1 (1.4)		2 (2.8)	1 (1.4)	

### Correlation of miR-1246 expression in various body fluids with ESCC prognosis

**Fig 3:**
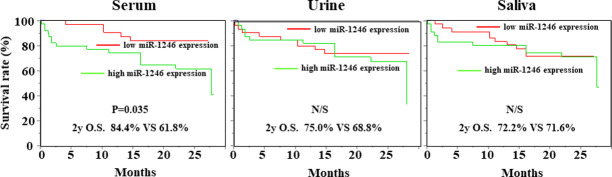
The prognostic value of the miR-1246 expression levels in various body fluids. The Kaplan–Meier analysis and log-rank test showed that there was a significant difference (P = 0.035) between patients with higher and lower levels of serum miR-1246 expression in overall survival. On the other hand, there was no correlation between the expression level of miR-1246 in urine or saliva samples and prognosis.

Overall survival analysis was performed using the Kaplan–Meier approach, with statistical analysis performed using the log-rank test. Using the patient groups described above, the prognostic value of miR-1246 expression was examined. There were no significant differences in the survival of the patients in the high- and low-expression groups based on the data for the body fluids other than serum. The prognosis of the group with high serum miR-1246 expression was significantly worse than that of the group with low serum miR-1246 expression (p = 0.035), consistent with our previous report [19]. The 2-year survival rates for the high and low serum miR-1246 expression groups were 84.4% and 61.8%, respectively (Fig 3).

### Postoperative miR-1246 expression levels in each body fluid

**Fig 4:**
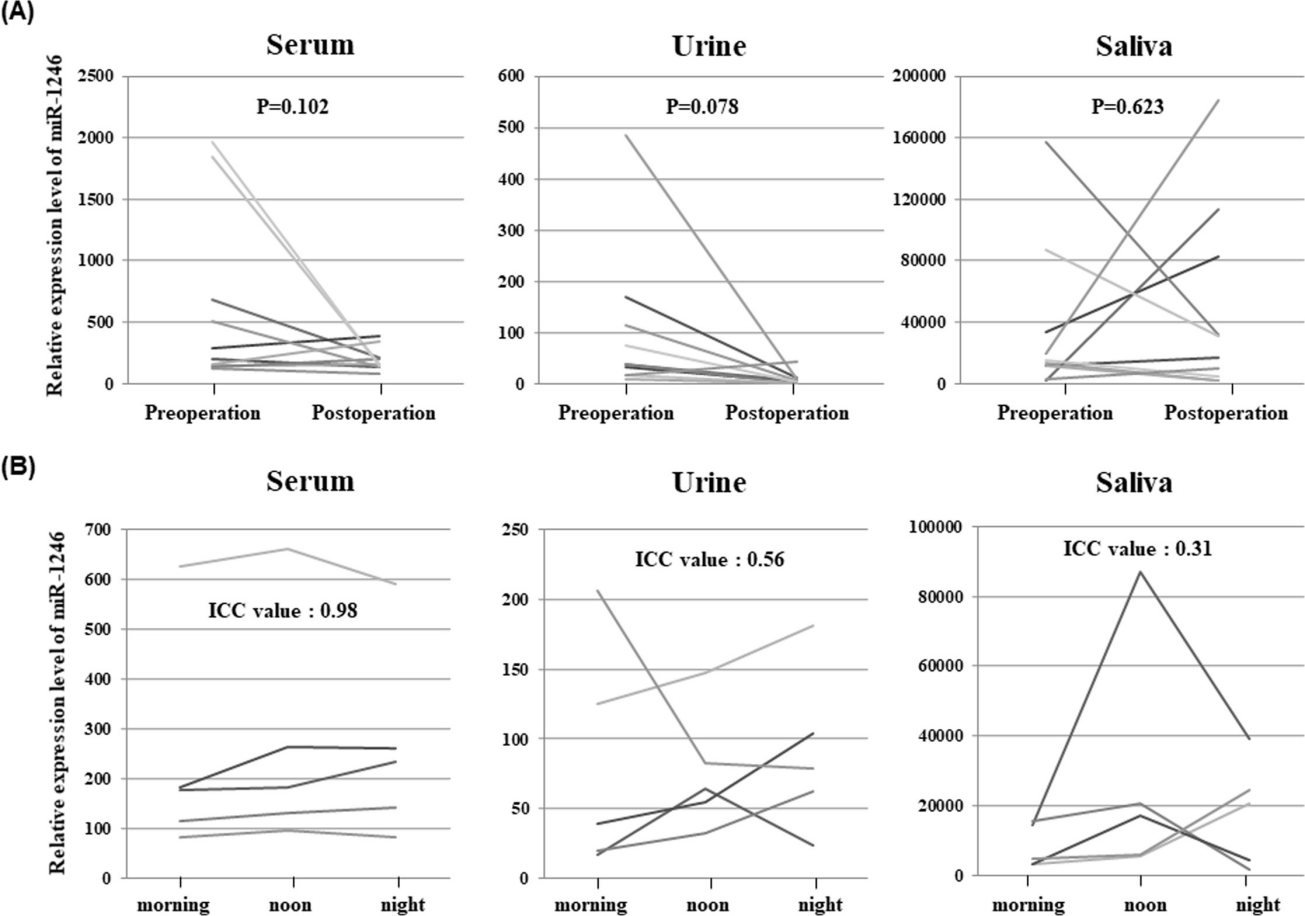
A. The results of a comparison of the serum miR-1246 expression levels between pre- and postoperative samples in various body fluids. In serum and urine, miR-1246 expression was confirmed to be reduced postoperatively in many cases but was not statistically significant. On the other hand, the expression level of miR-1246 in saliva did not show a clear decrease after operation. B. To evaluate the diurnal variation of miR-1246 in various body fluids, the intraclass correlation coefficient (ICC) was calculated using a two-way random model. MiR-1246 expression was examined in each body fluid three times in one day (in the morning, at noon, and at night), and varied greatly between time points in non-serum samples. ICC values for miR1246 in blood, urine and sputum were 0.98, 0.56 and 0.31.

In 10 cases, the expression of miR-1246 in each body fluid was evaluated pre- and post-operation. In the serum and urine, miR-1246 expression was confirmed to be reduced postoperatively in many cases, but the differences were not statistically significant (Fig 4A). Meanwhile, saliva did not show any obvious decrease in postoperative miR-1246 expression.

### Changes in miR-1246 expression in different body fluids over time

The expression of miR-1246 was examined in each body fluid three times in one day (in the morning, at noon, and at night) and was found to vary greatly between the time points in non-serum samples. The ICC values for miR-1246 were 0.98, 0.56, and 0.31 in the blood, urine, and sputum, indicating an almost perfect, moderate, and fair agreement, respectively (Fig 4B).

## Discussion

In this study, miR-1246 levels, not only in the serum but also in the urine and saliva, displayed a certain diagnostic capability for ESCC, with urinary and salivary miR-1246 levels displaying similar positivity rates as that of serum miR-1246. However, the correlations between miR-1246 levels and both clinicopathological factors and prognosis were diminished in non-serum samples. In particular, salivary levels varied widely throughout the day, which may have been the cause of their decreased sensitivity and specificity.

Serum miRs are expected to be valuable biomarkers and have been the focus of many recent reports. However, blood collection is invasive and should usually be performed in a medical institution. By contrast, urine and saliva can be collected at home, completely noninvasively. If proven useful as blood substitutes, urine and saliva could be used for clinical biomarker detection in the future. MicroRNA profiling of human embryonic stem cells obtained before and after differentiation into embryoid bodies revealed the sequences and expression levels of 334 known and 104 novel miRs, including miR-1246 [24]. We have previously reported that serum miR-1246 levels have diagnostic value in patients with ESCC [19]. Many other studies have also reported the utility of serum miR-1246 as a biomarker. In a study of high-grade serous ovarian cancer, serum miR-1246 expression was compared between 168 patients and 65 healthy controls, and cancer was detected with a sensitivity of 87% and a specificity of 77% [25]. In a study of liver tumors, increased serum miR-1246 expression was detected in 77% of patients with metastatic liver tumors and in 45% of patients with primary hepatocellular carcinoma [26]. Examination of exosomal miR-1246 expression in the serum of patients with early gastric cancer revealed that patients with stage I gastric cancer could be distinguished from healthy controls and patients with benign disease, with AUC values of 0.843 and 0.811, respectively [27]. In addition, a systematic review reported that miR-1246 was more useful than miR-21 or miR-4644 [28]. Therefore, serum miR-1246 may be a promising clinical biomarker for patients with cancer. A study of non-small cell lung cancer showed that miR-1246 conferred tumorigenicity and was required for lung cancer metastasis [29]. Mechanistically, inhibition of miR-1246 expression reduces stemness and epithelial–mesenchymal transition in non-small cell lung cancer, in addition to suppressing proliferation, sphere formation, colony formation, and invasion of tumor cells [30].

In addition to their presence in the blood, circulating miRs are present in several other body fluids, including urine, saliva, and cerebrospinal fluid. These small molecules are relatively stable and can be detected in association with particles that do not contain intracellular vesicles (usually protein complexes) or packaged in microvesicles or exosomes [31]. In this study, we examined miR-1246 levels in urine and saliva and found that their sensitivity of ESCC detection was equivalent to that of miR-1246 levels in the blood. In recent years, the number of reports on the usefulness of urine miRs has been increasing; however, most of these reports have studied urinary cancer, with the goal of measuring miRs directly secreted from the tumor into urine. In a rare nonurinary cancer case, a study of triple-negative breast cancer reported that miRs found in cancer-associated miR-17-92 clusters, as well as serum miRs, had reduced expression levels in the urine [32].

Based on the assumption that blood-derived molecules flow into salivary gland tissues via passive intracellular diffusion and active transport and enter salivary gland tissues via cellular mechanisms such as paracellular pathways, miR expression in the serum and saliva is thought to be similar [33,34]. To date, 18 salivary miRs (miR-1246, miR-4644, miR-21, miR-34a, mir-155, miR-200b, miR-376a, miR-23a, miR-23b, miR-29c, miR-210, miR-216, miR -940, miR-3679-5p, miR-17, miR-18b, miR-18a, and miR-196a) have been studied in gastrointestinal cancers and pancreatic cancer, regardless of tumor progression [35–39]. Similar to our study, Machida and colleagues [35] focused on miR-1246, and their ROC curve had an AUC of 0.814.

However, the reproducibility of data on miR levels in non-serum body fluids can be a problem. In contrast to that of blood, production of urine and saliva can vary greatly throughout the day. Saliva is a mixture of liquids that are produced and ultimately integrated by a number of glandular structures, including the parotid, submandibular, sublingual, and minor salivary glands, as well as the gingival sulcus [32]. Therefore, the quantity and quality of saliva may be greatly affected by factors such as the age, sex, circadian rhythm, diet, drugs, and environmental exposure [40]. Consequently, relative salivary miR expression levels may vary greatly. In this study, we examined variations in miR-1246 levels in the blood, urine, and saliva at various time points in a single day for the first time. Although the differences were not statistically significant, the results confirmed large fluctuations in saliva miR-1246 levels at various collection times. However, as expected, there were no significant changes in the serum levels between the time points. Depending on the threshold value used, accurate screening using miR levels in urine or saliva may be possible; however, our results indicate that the other two fluids are not superior to the blood.

A limitation of this study is that it was conducted at a single institution and with a relatively small sample size. In addition, the study focused on a single miR, while there are thousands of others that may be more useful biomarkers. We started implementing next-generation sequencing to identify useful miRs in the serum, urine, and saliva. In addition, we are in the process of developing a panel containing multiple miRs, which could be used as a kit in clinical practice.

## Conclusions

Our results showed that the expression levels of miR-1246 in body fluids other than the blood may be used instead of the serum miR-1246 levels as a diagnostic biomarker for ESCC in patients. Urine collection is noninvasive and can be performed anywhere, and our results support the use of urine samples instead of blood samples.

## Supporting information

S1 Data PCR result. (XLSX)

## References

[1] Bray F, Ferlay J, Soerjomataram I, Siegel RL, Torre LA, Jemal A. Global cancer statistics 2018: GLOBOCAN estimates of incidence and mortality worldwide for 36 cancers in 185 countries. (1542–4863 (Electronic)). 10.3322/caac.21492 30207593

[2] Brown LM, Devesa SS, Chow WH. Incidence of adenocarcinoma of the esophagus among white Americans by sex, stage, and age. J Natl Cancer Inst. 2008;100(16):1184–7. 10.1093/jnci/djn211 18695138 PMC2518165

[3] Ohashi S, Miyamoto S, Kikuchi O, Goto T, Amanuma Y, Muto M. Recent Advances From Basic and Clinical Studies of Esophageal Squamous Cell Carcinoma. Gastroenterology. 2015;149(7):1700–15. 10.1053/j.gastro.2015.08.054 26376349

[4] Kamangar F, Dores GM, Anderson WF. Patterns of cancer incidence, mortality, and prevalence across five continents: defining priorities to reduce cancer disparities in different geographic regions of the world. J Clin Oncol. 2006;24(14):2137–50. 10.1200/JCO.2005.05.2308 16682732

[5] Law S, Wong J. The current management of esophageal cancer. Adv Surg. 2007;41:93–119. 10.1016/j.yasu.2007.05.007 17972559

[6] Headrick JR, Nichols FC 3rd, Miller DL, Allen MS, Trastek VF, Deschamps C, et al. High-grade esophageal dysplasia: long-term survival and quality of life after esophagectomy. Ann Thorac Surg. 2002;73(6):1697–702; discussion 702–3. 10.1016/s0003-4975(02)03496-3 12078755

[7] Lin DC, Hao JJ, Nagata Y, Xu L, Shang L, Meng X, et al. Genomic and molecular characterization of esophageal squamous cell carcinoma. Nat Genet. 2014;46(5):467–73. 10.1038/ng.2935 24686850 PMC4070589

[8] Integrated genomic characterization of oesophageal carcinoma. Nature. 2017;541(7636):169–75. 10.1038/nature20805 28052061 PMC5651175

[9] Lu J, Getz G, Miska EA, Alvarez-Saavedra E, Lamb J, Peck D, et al. MicroRNA expression profiles classify human cancers. Nature. 2005;435(7043):834–8. 10.1038/nature03702 15944708

[10] Lawrie CH, Gal S, Dunlop HM, Pushkaran B, Liggins AP, Pulford K, et al. Detection of elevated levels of tumour-associated microRNAs in serum of patients with diffuse large B-cell lymphoma. Br J Haematol. 2008;141(5):672–5. 10.1111/j.1365-2141.2008.07077.x 18318758

[11] Mitchell PS, Parkin RK, Kroh EM, Fritz BR, Wyman SK, Pogosova-Agadjanyan EL, et al. Circulating microRNAs as stable blood-based markers for cancer detection. Proc Natl Acad Sci U S A. 2008;105(30):10513–8. 10.1073/pnas.0804549105 18663219 PMC2492472

[12] Chen X, Ba Y, Ma L, Cai X, Yin Y, Wang K, et al. Characterization of microRNAs in serum: a novel class of biomarkers for diagnosis of cancer and other diseases. Cell Res. 2008;18(10):997–1006. 10.1038/cr.2008.282 18766170

[13] Srivastava A, Moxley K, Ruskin R, Dhanasekaran DN, Zhao YD, Ramesh R. A Non-invasive Liquid Biopsy Screening of Urine-Derived Exosomes for miRNAs as Biomarkers in Endometrial Cancer Patients. Aaps j. 2018;20(5):82. 10.1208/s12248-018-0220-y 29987691

[14] Kao HW, Pan CY, Lai CH, Wu CW, Fang WL, Huang KH, et al. Urine miR-21-5p as a potential non-invasive biomarker for gastric cancer. Oncotarget. 2017;8(34):56389–97. 10.18632/oncotarget.16916 28915598 PMC5593569

[15] Zhou J, Gong G, Tan H, Dai F, Zhu X, Chen Y, et al. Urinary microRNA-30a-5p is a potential biomarker for ovarian serous adenocarcinoma. Oncology reports. 2015;33(6):2915–23. 10.3892/or.2015.3937 25962395

[16] Rapado-González Ó, Majem B, Álvarez-Castro A, Díaz-Peña R, Abalo A, Suárez-Cabrera L, et al. A Novel Saliva-Based miRNA Signature for Colorectal Cancer Diagnosis. J Clin Med. 2019;8(12). 10.3390/jcm8122029 31757017 PMC6947363

[17] Salazar-Ruales C, Arguello JV, López-Cortés A, Cabrera-Andrade A, García-Cárdenas JM, Guevara-Ramírez P, et al. Salivary MicroRNAs for Early Detection of Head and Neck Squamous Cell Carcinoma: A Case-Control Study in the High Altitude Mestizo Ecuadorian Population. BioMed research international. 2018;2018:9792730. 10.1155/2018/9792730 30584540 PMC6280231

[18] Li F, Yoshizawa JM, Kim KM, Kanjanapangka J, Grogan TR, Wang X, et al. Discovery and Validation of Salivary Extracellular RNA Biomarkers for Noninvasive Detection of Gastric Cancer. Clin Chem. 2018;64(10):1513–21. 10.1373/clinchem.2018.290569 30097497 PMC7720197

[19] Takeshita N, Hoshino I, Mori M, Akutsu Y, Hanari N, Yoneyama Y, et al. Serum microRNA expression profile: miR-1246 as a novel diagnostic and prognostic biomarker for oesophageal squamous cell carcinoma. British journal of cancer. 2013;108(3):644–52. 10.1038/bjc.2013.8 23361059 PMC3593570

[20] Zhang Y, Liao JM, Zeng SX, Lu H. p53 downregulates Down syndrome-associated DYRK1A through miR-1246. EMBO Rep. 2011;12(8):811–7. 10.1038/embor.2011.98 21637297 PMC3147276

[21] Xu YF, Hannafon BN, Zhao YD, Postier RG, Ding WQ. Plasma exosome miR-196a and miR-1246 are potential indicators of localized pancreatic cancer. Oncotarget. 2017;8(44):77028–40. 10.18632/oncotarget.20332 29100367 PMC5652761

[22] Ishige F, Hoshino I, Iwatate Y, Chiba S, Arimitsu H, Yanagibashi H, et al. MIR1246 in body fluids as a biomarker for pancreatic cancer. Scientific reports. 2020;10(1):8723. 10.1038/s41598-020-65695-6 32457495 PMC7250935

[23] Landis JR, Koch GG. The measurement of observer agreement for categorical data. Biometrics. 1977;33(1):159–74. 843571

[24] Morin RD, O’Connor MD, Griffith M, Kuchenbauer F, Delaney A, Prabhu AL, et al. Application of massively parallel sequencing to microRNA profiling and discovery in human embryonic stem cells. Genome Res. 2008;18(4):610–21. 10.1101/gr.7179508 18285502 PMC2279248

[25] Todeschini P, Salviato E, Paracchini L, Ferracin M, Petrillo M, Zanotti L, et al. Circulating miRNA landscape identifies miR-1246 as promising diagnostic biomarker in high-grade serous ovarian carcinoma: A validation across two independent cohorts. Cancer Lett. 2017;388:320–7. 10.1016/j.canlet.2016.12.017 28017893

[26] Ahmed EK, Fahmy SA, Effat H, Wahab AHA. Circulating MiR-210 and MiR-1246 as Potential Biomarkers for Differentiating Hepatocellular Carcinoma from Metastatic Tumors in the Liver. Journal of medical biochemistry. 2019;38(2):109–17. 10.2478/jomb-2018-0010 30867638 PMC6411000

[27] Shi Y, Wang Z, Zhu X, Chen L, Ma Y, Wang J, et al. Exosomal miR-1246 in serum as a potential biomarker for early diagnosis of gastric cancer. International journal of clinical oncology. 2020;25(1):89–99. 10.1007/s10147-019-01532-9 31506750

[28] Wei C, Li Y, Huang K, Li G, He M. Exosomal miR-1246 in body fluids is a potential biomarker for gastrointestinal cancer. Biomarkers in medicine. 2018;12(10):1185–96. 10.2217/bmm-2017-0440 30235938

[29] Zhang WC, Chin TM, Yang H, Nga ME, Lunny DP, Lim EK, et al. Tumour-initiating cell-specific miR-1246 and miR-1290 expression converge to promote non-small cell lung cancer progression. Nat Commun. 2016;7:11702. 10.1038/ncomms11702 27325363 PMC4919505

[30] Kim G, An HJ, Lee MJ, Song JY, Jeong JY, Lee JH, et al. Hsa-miR-1246 and hsa-miR-1290 are associated with stemness and invasiveness of non-small cell lung cancer. Lung Cancer. 2016;91:15–22. 10.1016/j.lungcan.2015.11.013 26711929

[31] Sanz-Rubio D, Martin-Burriel I, Gil A, Cubero P, Forner M, Khalyfa A, et al. Stability of Circulating Exosomal miRNAs in Healthy Subjects. Scientific reports. 2018;8(1):10306. 10.1038/s41598-018-28748-5 29985466 PMC6037782

[32] Ritter A, Hirschfeld M, Berner K, Rücker G, Jäger M, Weiss D, et al. Circulating non‑coding RNA‑biomarker potential in neoadjuvant chemotherapy of triple negative breast cancer? Int J Oncol. 2020;56(1):47–68. 10.3892/ijo.2019.4920 31789396 PMC6910196

[33] Yoshizawa JM, Schafer CA, Schafer JJ, Farrell JJ, Paster BJ, Wong DT. Salivary biomarkers: toward future clinical and diagnostic utilities. Clinical microbiology reviews. 2013;26(4):781–91. 10.1128/CMR.00021-13 24092855 PMC3811231

[34] Streckfus CF, Mayorga-Wark O, Arreola D, Edwards C, Bigler L, Dubinsky WP. Breast cancer related proteins are present in saliva and are modulated secondary to ductal carcinoma in situ of the breast. Cancer investigation. 2008;26(2):159–67. 10.1080/07357900701783883 18259946

[35] Machida T, Tomofuji T, Maruyama T, Yoneda T, Ekuni D, Azuma T, et al. miR1246 and miR4644 in salivary exosome as potential biomarkers for pancreatobiliary tract cancer. Oncology reports. 2016;36(4):2375–81. 10.3892/or.2016.5021 27573701

[36] Alemar B, Izetti P, Gregorio C, Macedo GS, Castro MA, Osvaldt AB, et al. miRNA-21 and miRNA-34a Are Potential Minimally Invasive Biomarkers for the Diagnosis of Pancreatic Ductal Adenocarcinoma. Pancreas. 2016;45(1):84–92. 10.1097/MPA.0000000000000383 26262588

[37] Humeau M, Vignolle-Vidoni A, Sicard F, Martins F, Bournet B, Buscail L, et al. Salivary MicroRNA in Pancreatic Cancer Patients. PloS one. 2015;10(6):e0130996. 10.1371/journal.pone.0130996 26121640 PMC4486170

[38] Xie Z, Yin X, Gong B, Nie W, Wu B, Zhang X, et al. Salivary microRNAs show potential as a noninvasive biomarker for detecting resectable pancreatic cancer. Cancer prevention research (Philadelphia, Pa). 2015;8(2):165–73. 10.1158/1940-6207.CAPR-14-0192 25538087

[39] Gao S, Chen LY, Wang P, Liu LM, Chen Z. MicroRNA expression in salivary supernatant of patients with pancreatic cancer and its relationship with ZHENG. BioMed research international. 2014;2014:756347. 10.1155/2014/756347 25126577 PMC4122139

[40] Humphrey SP, Williamson RT. A review of saliva: normal composition, flow, and function. The Journal of prosthetic dentistry. 2001;85(2):162–9. 10.1067/mpr.2001.113778 11208206

